# Investigation of the degree of family history of diabetes in different clusters of newly diagnosed type 2 diabetes in Thailand

**DOI:** 10.1080/07853890.2025.2500697

**Published:** 2025-05-08

**Authors:** Watip Tangjittipokin, Tassanee Narkdontri, Nipaporn Teerawattanapong, Sarocha Suthon, Vorthunju Nakhonsri, Rujipat Wasitthankasem, Nirinya Sudtachat, Lukana Preechasuk, Varisara Lapinee, Nuntakorn Thongtang, Sissades Tongsima, Nattachet Plengvidhya

**Affiliations:** a Department of Immunology, Faculty of Medicine Siriraj Hospital, Mahidol University, Bangkok, Thailand; b Siriraj Center of Research Excellence for Diabetes and Obesity, Faculty of Medicine Siriraj Hospital, Mahidol University, Bangkok, Thailand; c Research Department, Faculty of Medicine Siriraj Hospital, Mahidol University, Bangkok, Thailand; d National Biobank of Thailand, National Science and Technology Development Agency, Khlong Nueng, Thailand; e Siriraj Diabetes Center of Excellence, Faculty of Medicine Siriraj Hospital, Mahidol University, Bangkok, Thailand; f Division of Endocrinology and Metabolism, Department of Medicine, Faculty of Medicine Siriraj Hospital, Mahidol University, Bangkok, Thailand

**Keywords:** Type 2 diabetes, genetic risk factor, age at diagnosis, family history, degree relative

## Abstract

**Aim:**

Type 2 diabetes is a heterogeneous disease with strong genetic components. We showed earlier that newly diagnosed type 2 diabetes in Thai patients could be categorized into four clusters. This study aimed to determine the evidence of hereditary factors in these type 2 diabetes clusters.

**Methods:**

A total of 487 subjects who were diagnosed with type 2 diabetes in two years were enrolled in the Siriraj Diabetes Center, Siriraj Hospital Bangkok, Bangkok, Thailand. They were divided into four clusters as previously described. The associations between patients’ characteristics, degree of family history of diabetes (FHD), and type 2 diabetes clusters were tested using multinomial logistic regression.

**Results:**

Among four clusters of newly diagnosed type 2 diabetes, there were significant differences in characteristics at baseline, including age at diagnosis, BMI, waist circumference, blood sugar levels, vital signs, triglyceride, HDL, calculated LDL, creatinine and eGFR (all *p* < .05). A relatively young age at the time of diabetes diagnosis was associated with having second-degree relatives with diabetes (*p* < .05) in all clusters when using mild age-related diabetes (MARD) cluster with no FHD as a control. Patients in the severe insulin-deficient diabetes (SIDD) cluster had more first-degree relatives with diabetes (odds ratio = 1.85; *p* = .0354), while patients in the metabolic syndrome diabetes (MSD) cluster (odds ratio = 10.73; *p* < .001) and the mild obesity-related diabetes (MOD) group (odds ratio = 6.66; *p* = .002), had more second-degree relatives with diabetes.

**Conclusions:**

Genetic factors might have various roles in the pathogenesis of type 2 diabetes, at least in newly diagnosed Thai patients. Our findings supported that genetic heterogeneity contributed to clinical heterogeneity or four different clusters. Further studies are needed in a larger sample size of these patients is needed to identify genetic loci associated with each cluster.

## Introduction

Type 2 diabetes is increasing. The global prevalence of the disease in 2019 was estimated at 9.3% (463 million people) and will reach 10.9% (700 million people) in 2045 [1,2]. In Southeast Asia, the International Diabetes Foundation (IDF) reported that the number of people with diabetes will increase by 68%, reaching 152 million by 2045. More than 90% of diabetes cases globally are type 2 diabetes [3].

Type 2 diabetes is a heterogeneous disease resulting from the interaction between genes and environmental factors [4]. Several large-scale genome-wide association studies (GWASs) have identified hundreds of loci associated with the disease among ethnicities [5]. Nevertheless, only in South Asian T2D cases that 21 novel genetic loci were identified after cross-ancestry comparisons (East Asian, African and Hispanic populations) [6]. Therefore, it is well established that type 2 diabetes has genetic heterogeneity. Recently, many research groups, including ours, have shown that type 2 diabetes has also clinical heterogeneity since diagnosis [7–11]. In Thai patients with newly diagnosed type 2 diabetes, four clusters were identified: cluster 1 (severe insulin-deficient diabetes, SIDD), cluster 2 (metabolic syndrome diabetes, MSD), cluster 3 (mild obesity-related diabetes, MOD) and cluster 4 (mild age-related diabetes, MARD) [7]. It is possible that different genetic factors were associated with different type 2 diabetes clusters.

We previously categorized newly diagnosed Thai type 2 diabetes into four clusters based on age at diagnosis, baseline BMI, HbA1c, triglycerides (TGs) and HDL [7]. Each cluster had dissimilar disease progression and the development of chronic complications [7]. Genetic factors are one of the main determinants of type 2 diabetes. The objective of this study was to investigate whether there is a genetic heterogeneity between different clusters of newly diagnosed type 2 diabetes in Thailand by determining the associations between patients’ characteristics, degree of family history of diabetes (FHD), and type 2 diabetes clusters.

## Materials and methods

### Subjects, data collection and measurements

Four hundred and eighty-seven participants were enrolled at Siriraj Diabetes Center (SDC) as part of the Siriraj diabetes registry (which has been operating since 2015), Siriraj Hospital, Bangkok, Thailand. The patients with type 2 diabetes were diagnosed according to the American Diabetes Association (ADA) guidelines [4]. The registry has been in operation since February 2015. All patients in this study were recruited from 2022 to 2024. The inclusion criteria were patients age ≥18 years old, diagnosed with type 2 diabetes according to the ADA criteria [4] within two years of registration, fasting plasma glucose (FPG) ≥7.0 mmol/L and HbA1c ≥48 mmol/mol. The exclusion criteria were patients diagnosed with other types of diabetes, patients with congestive heart failure, severe liver impairment, end stage renal disease, advance stage of cancer or terminally ill. Age at diagnosis, sex assigned at birth, BMI, waist circumference (W), systolic blood pressure (SBP), diastolic blood pressure (DBP) and pulse rate (PR) were collected. Laboratory measurements included total cholesterol (TC), TG, HDL, LDL, creatinine (Cr) and estimated glomerular filtration rate (eGFR). Measurement methods were previously described [7]. The research was carried out according to the Declaration of Helsinki and informed consent was written and obtained from the patients when appropriate. The entire study was approved by the Siriraj Institutional Review Board (SIRB), Siriraj Hospital Faculty of Medicine, Mahidol University, Bangkok, Thailand (COA no. Si 826/2022).

### Classification of the degree of family history of diabetes

The degree of FHD was classified into four groups: (I) no relatives with diabetes, (II) only one first-degree relative with diabetes, (III) ≥2 relatives with diabetes with ≥1 first-degree relative with diabetes and (IV) ≥1-second degree relative with diabetes but without first degree relative with diabetes.

### Clusters of Thai patients with newly diagnosed type 2 diabetes

K-means analysis was used to classify patients into subgroups using five variables, including age at diagnosis, baseline BMI, HbA1c, TG and HDL-C. Continuous measures were mean-centred and standardized. Continuous measures greater than 5 S.D. from the mean were excluded. The first step of clustering was to estimate the optimal number of clusters based on the silhouette width and the elbow method. After clustering using five variables, the similarity was checked within each cluster, and the difference between the clusters using a three-dimensional plot. Robust tests of equal means (*p* < .001) and the Games-Howell post hoc test were performed. Clustering was defined using five variables, including age at diagnosis, BMI, HbA1c, TG and HDL. There were a total of four clusters [7]:

*Cluster 1* (SIDD): high HbA1c and low BMI.
*Cluster 2* (MSD): high TG, low HDL, average age and BMI.
*Cluster 3* (MOD): high BMI, lower HbA1c and young age.
*Cluster 4* (MARD): older age and relatively lower HbA1c at diagnosis.

### Statistical analysis

All statistical analyses were performed using R (R Foundation for Statistical Computing, Vienna, Austria), a free software environment for statistical computing and graphics (http://www.r-project.org). Two-sided *p* values <.05 were considered statistically significant. Baseline descriptive data were summarized as mean ± standard deviation (mean ± S.D.) for continuous variables and as frequencies (number and percentage) for categorical variables. Comparisons of baseline variables across different clusters or family history groups were conducted using one-way ANOVA for normally distributed data or the Kruskal–Wallis test for non-normally distributed data, as appropriate. The distribution of categorical variables, such as the type 2 diabetes clusters by FHD, was examined using Pearson’s Chi-square test or Fisher’s exact test.

Multinomial logistic regression was used to assess the association between type 2 diabetes clusters and the degree of FHD, using the MARD cluster as the reference group. Before model fitting, key statistical assumptions were tested, including independence of observations, absence of multicollinearity (assessed using variance inflation factor (VIF)) and linearity of continuous predictors in the logit scale. All assumptions were met, supporting the validity of regression analyses.

## Results

### Baseline characteristics of four newly diagnosed type 2 diabetes clusters

Four hundred and eighty-seven participants were divided into four clusters: cluster 1 SIDD (*n* = 93, 19%), cluster 2 MSD (*n* = 55, 11%), cluster 3 MOD (*n* = 119, 25%) and cluster 4 MARD (*n* = 220, 45%). Demographic data and blood chemistry analysis showed that age at diagnosis, BMI, waist circumference, blood pressure, FPG, HbA1c, TG, HDL, Cr and eGFR level were significant differences among clusters (*p* < .05 and *p* < .001). MARD had the highest age at the time of diabetes diagnosis. As expected, MSD had obvious features of metabolic syndrome (higher SBP, higher TG but lower HDL level). SIDD had the poorest glycaemic control probably due to severe insulin deficiency (Table 1). There were no significant differences in gender and direct LDL levels between all clusters.

**Table 1.** Baseline clinical characteristics and laboratory parameters of the enrolled patients.

	Cluster 1 SIDD (*n* = 93)	Cluster 2 MSD (*n* = 55)	Cluster 3 MOD (*n* = 119)	Cluster 4 MARD (*n* = 220)	*p* Value
*Demographic*					
Age at diagnosis (years)	52.33 ± 8.51	48.84 ± 11.16	40.78 ± 7.6	60.42 ± 6.94	<.001**
Male (%)	48.39	47.27	31.93	40.91	.07
BMI (kg/m^2^)	24.94 ± 3.93	27.57 ± 4.7	32.58 ± 6.5	26.24 ± 4.04	<.001**
Waist circumference (cm)	85.28 ± 7.83	90.52 ± 10.55	99.73 ± 18.75	90.46 ± 9.97	<.001**
*Blood sugar*					
Fasting plasma glucose (mmol/L)	9.90 ± 4.27	9.10 ± 3.63	8.83 ± 2.72	7.49 ± 1.65	<.001**
HbA1c (mmol/mol)	87 ± 10	68 ± 3.7	63.1 ± 4.4	52.0 ± 11.7	<.001**
*Vital sign*					
Systolic blood pressure (mm Hg)	126.87 ± 16.76	132.4 ± 15.07	131.66 ± 13.5	131.5 ± 14.07	.041*
Diastolic blood pressure (mm Hg)	74.43 ± 11.73	77.78 ± 11.83	80.26 ± 11.16	74.65 ± 10.88	<.001**
Pulse rate (BPM)	88.29 ± 12.23	88.67 ± 14.65	89.48 ± 13.43	82.05 ± 12.91	<.001**
*Blood chemistry*					
Triglyceride (mmol/L)	1.45 ± 0.55	3.36 ± 1.43	1.72 ± 0.61	1.54 ± 0.69	<.001**
HDL (mmol/L)	1.32 ± 0.30	1.07 ± 0.28	1.20 ± 0.28	1.43 ± 0.35	<.001**
Calculated LDL (mmol/L)	3.00 ± 1.08	2.48 ± 1.20	2.80 ± 1.00	2.63 ± 0.93	.013*
Direct LDL (mmol/L)	3.02 ± 0.89	3.23 ± 1.03	3.00 ± 1.10	3.18 ± 0.76	.79
Creatinine (mmol/L)	0.07 ± 0.02	0.07 ± 0.03	0.06 ± 0.02	0.07 ± 0.02	<.001**
eGFR (mL/min/1.73 m^2^)	96.11 ± 18.13	95.73 ± 21.35	106.43 ± 15.16	85.29 ± 16.88	<.001**

eGFR: estimated glomerular filtration rate; MARD: mild age-related diabetes; SIDD: severe insulin-deficient diabetes; MSD: metabolic syndrome diabetes; MOD: mild obesity-related diabetes.

The data presented in mean ± standard deviation (S.D.), number of cases, and percentage. The bold text of *p* value is considered statistically significant (*p < .05 and **p < .001).

### The correlation between clinical characteristics and laboratory parameters of patients and clusters of newly diagnosed type 2 diabetes

**Figure 1.**
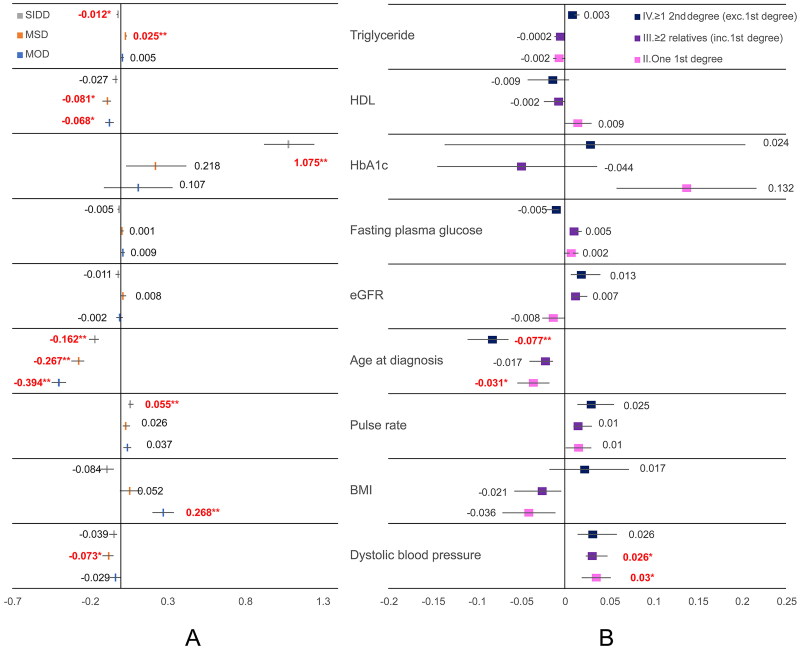
Correlation among baseline clinical characteristics and laboratory parameters with (A) type 2 diabetes clusters and (B) degree of family history of diabetes relatives with family diabetes.

The correlation between variables and four clusters in type 2 diabetes was determined using multinomial logistic regression analysis, with the MARD cluster used as a reference (Figure 1(A)). The beta coefficient in the SIDD cluster was significantly associated with HbA1c (Beta = 1.075, *p* = 3.188E − 11), PR (Beta = 0.055, *p* = .001) and TG (Beta = −0.012, *p* = .007). For MSD, the variables were significantly related to TG (Beta = 0.025, *p* = 1.691E − 09), HDL (Beta = −0.081, *p* = .003) and DBP (Beta = −0.073, *p* = .040). Furthermore, HDL and BMI levels were significantly associated with the MOD cluster (Beta = −0.068, *p* = .002 and Beta = 0.268, *p* = 2.577E − 05, respectively). Age at diagnosis of type 2 diabetes was significantly associated with the SIDD cluster (Beta = −0.162, *p* = 2.780E − 07), MSD cluster (Beta = −0.267, *p* = 1.265E − 10) and the MOD cluster (Beta = −0.394, *p* = 2.529E − 22).

### The association between the degree of family history of diabetes and the clusters of newly diagnosed type 2 diabetes

**Figure 2.**
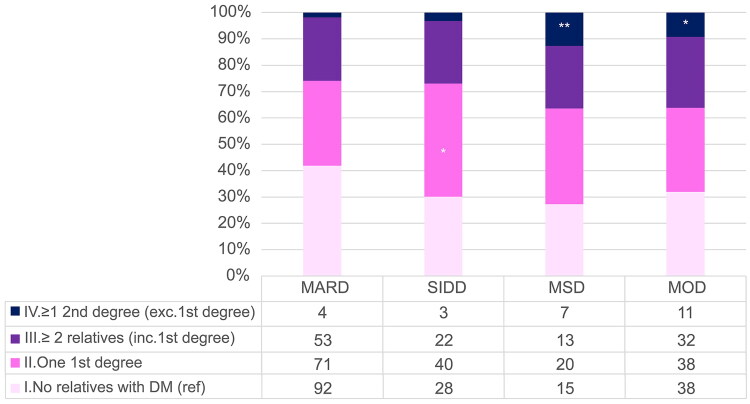
Distribution of different degrees of family history of diabetes among each type 2 diabetes cluster.

The association between each type 2 diabetes cluster and the degree of FHD was analysed using MARD and no relative with diabetes (I) as reference (Table 2 and Figure 2). SIDD was significantly associated with one first-degree relative with diabetes (II) (odds ratio = 1.85, 95% CI: 1.043–3.285, *p* = .0354) and the number of patients in this category was 43.01%. MSD and MOD were significantly associated with ≥1 s degree relative to diabetes but no first degree relative to diabetes (IV) (MSD: odds ratio = 10.73, 95% CI: 2.799–41.165, *p* = .0005 and MOD: odds ratio = 6.66, 95% CI: 1.995–22.220, *p* = .002). Notably, the percentages of patients in this category of MSD and MOD were 12.73% and 9.24%, respectively.

**Table 2.** The association among T2D clusters and the degree of family history of diabetes.

	MARD (ref.) *N* = 220	SIDD (*N* = 93)			MSD (*N* = 55)			MOD (*N* = 119)		
Degree of family history of diabetes	%	%	Odds ratio (95% CI)	*p* Value	%	Odds ratio (95% CI)	*p* Value	%	Odds ratio (95% CI)	*p* Value
No relatives with DM (*N* = 173)	41.82 (*N* = 92)	30.11 (*N* = 28)	1	(ref.)	27.27 (*N* = 15)	1	(ref.)	31.93 (*N* = 38)	1	(ref.)
One 1st degree with DM (*N* = 169)	32.27 (*N* = 71)	43.01 (*N* = 40)	1.85 (1.043–3.285)	.0354*	36.36 (*N* = 20)	1.73 (0.826–3.612)	.1462	31.93 (*N* = 38)	1.3 (0.751–2.237)	.3522
≥2 relatives with DM (inc. 1st degree) (*N* = 120)	24.09 (*N* = 53)	23.66 (*N* = 22)	1.36 (0.710–2.620)	.3513	23.64 (*N* = 13)	1.5 (0.665–3.402)	.3266	26.89 (*N* = 32)	1.46 (0.819–2.608)	.1988
≥1 2nd degree DM (exc. 1st degree) (*N* = 25)	1.82 (*N* = 4)	3.23 (*N* = 3)	2.46 (0.520–11.670)	.2558	12.73 (*N* = 7)	10.73 (2.799–41.165)	.0005**^,a^	9.24 (*N* = 11)	6.66 (1.995–22.220)	.002*^,a^

DM: diabetes mellitus; MARD: mild age-related diabetes; SIDD: severe insulin-deficient diabetes; MSD: metabolic syndrome diabetes; MOD: mild obesity-related diabetes.

The data are presented in the number of cases, percentage, odd ratio and 95% CI (confidence interval). The bold text of the *p* value is considered statistically significant (*p < .05 and **p < .001). They were analysed using multinomial logistic regression, a reference as MARD, and no relatives with diabetes (^a^Fisher’s exact probability test for nonparametric test for categorical data with counts less than 5).

### The association between the degree of family history of diabetes and the age at diabetes diagnosis in clusters of newly diagnosed type 2 diabetes

**Figure 3.**
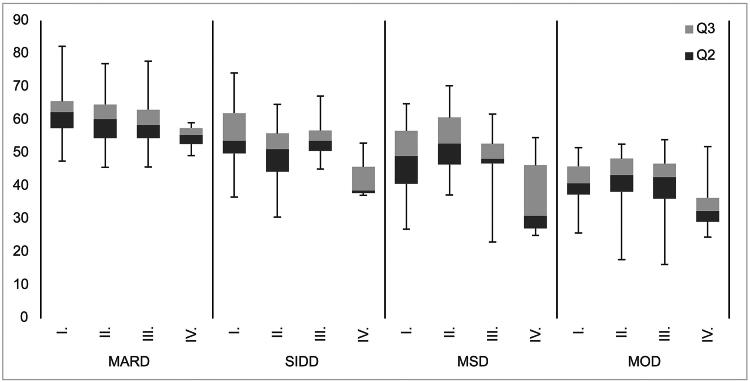
Association between age at diagnosis and degrees of family history of diabetes among each type 2 diabetes cluster.

The correlation between FHD and the age at diagnosis of diabetes in each type 2 diabetes cluster was analysed using no FHD (I) as a reference (Figure 3). The age at diagnosis in MARD was significantly younger in patients with a FHD (both first- and second-degree relatives) compared to those who had not (Figure 3). Meanwhile, younger age at diagnosis of SIDD and MOD was identified in patients with only a first degree relative with diabetes (II) and ≥1-second degree relative with diabetes but no first degree relative with diabetes (IV) (all *p* < .05). In MSD, a younger age at diagnosis was only in ≥1 s-degree relative with diabetes but no first-degree relative with diabetes (IV) (*p* < .05).

### Relationship between clinical characteristics and laboratory parameters of patients and the degree of family history of diabetes

To investigate the effects of genetics on all variables, the association between descriptive variables and the degree of FHD was analysed (Table 3). Baseline clinical variables were significantly different between the degree of FHD at the age of diabetes onset (*p* < .001), BMI (*p* = .006), FPG (*p* = .01), HbA1c (*p* = .01), PR (*p* = .044), Cr (*p* = .01) and eGFR (*p* < .001). Furthermore, type 2 diabetes clusters were associated with the degree of FHD (*p* = .006).

**Table 3.** Baseline clinical characteristics and laboratory parameters of enrolled patients vs. different degrees of family history of diabetes.

	No relatives with DM (*N* = 173)	One 1st degree with DM (*N* = 169)	≥2 relatives with DM (inc. 1st degree) (*N* = 120)	≥1 2nd degree with DM (exc. 1st degree) (*N* = 25)	*p* Value
*Baselines*					
Age at diagnosis (years)	55.07 ± 11.69	52.91 ± 10.05	52.07 ± 10.22	39.26 ± 11.38	<.001**
Male (%)	47.98	40.24	34.17	28.00	.06
BMI (kg/m^2^)	27.57 ± 5.41	27.35 ± 6.24	27.57 ± 4.41	31.19 ± 6.23	.006*
Waist circumference (cm)	92.14 ± 13.75	90.43 ± 14.82	92.07 ± 9.72	94.63 ± 9.53	.27
Fasting plasma glucose (mmol/L)	7.98 ± 2.92	8.75 ± 3.05	8.68 ± 2.91	8.67 ± 2.65	.01*
HbA1c (mmol/mol)	58.5 ± 0.9	66.9 ± 3.5	63.9 ± 0.3	66.4 ± 1.7	.01*
Systolic blood pressure (mm Hg)	130.61 ± 14.43	131.09 ± 15.3	130.02 ± 14.6	133.04 ± 12.91	.86
Diastolic blood pressure (mm Hg)	74.33 ± 10.81	77.49 ± 11.68	76.84 ± 11.38	79.92 ± 12.98	.08
Pulse rate (BPM)	83.76 ± 12.91	86.36 ± 13.88	86.81 ± 12.77	91.96 ± 16.53	.044*
Triglyceride (mmol/L)	1.76 ± 0.89	1.70 ± 0.94	1.80 ± 1.00	2.33 ± 1.28	.11
HDL (mmol/L)	1.30 ± 0.35	1.34 ± 0.37	1.31 ± 0.30	1.19 ± 0.29	.12
Calculated LDL (mmol/L)	2.70 ± 1.01	2.66 ± 1.01	2.86 ± 1.01	2.71 ± 1.03	.40
Direct LDL (mmol/L)	2.81 ± 0.78	3.34 ± 0.97	3.24 ± 0.88	N/A	.17
Creatinine (mmol/L)	0.07 ± 0.02	0.07 ± 0.02	0.07 ± 0.02	0.06 ± 0.01	.01*
eGFR (mL/min/1.73 m^2^)	90.06 ± 19.61	92.42 ± 19.37	96.78 ± 17.43	107 ± 16.02	<.001**
*T2D clusters*					
Cluster 1: SIDD (%, *n* = 93)	30.11	43.01	23.66	3.23	.006*^,t^
Cluster 2: MSD (%, *n* = 55)	27.27	36.36	23.64	12.73	
Cluster 3: MOD (%, *n* = 119)	31.93	31.93	26.89	9.24	
Cluster 4: MARD (%, *n* = 220)	41.82	32.27	24.09	1.82	

DM: diabetes mellitus; eGFR: estimated glomerular filtration rate; MARD: mild age-related diabetes; SIDD: severe insulin-deficient diabetes; MSD: metabolic syndrome diabetes; MOD: mild obesity-related diabetes.

Data presented in mean ± standard deviation (S.D.), number of cases, and percentage. The bold text of *p* value is considered statistically significant (*p < .05 and **p < .001). The category of variables was analysed using Pearson’s Chi-square test (t).

The association between the clinical characteristics of patients and the family history group in each T2D cluster is illustrated in Table 4. In the MARD cluster, we found that only the age of diabetes onset was significantly associated with family history (*p* < .001), with a trend for dLDL (*p* = .08). In the MOD cluster, age of diabetes onset FPG, and HbA1c were significantly associated with family history (*p* = .012, .004 and .007, respectively). For the MSD cluster, BMI, HbA1c, Cr and eGFR showed associations with family history (*p* = .043, <.001, .033 and .007, respectively). Finally, in the SIDD cluster, Cr and eGFR were associated with family history (*p* = .005 and .001, respectively).

**Table 4.** Clinical characteristics and laboratory parameters in different degrees of family history of diabetes clusters.

	No relatives with DM (*N* = 28)	One 1st degree DM (*N* = 40)	≥2 relatives with DM and ≥1 1st degree DM (*N* = 22)	≥1 2nd degree DM (*N* = 3)	*p* Value
*SIDD cluster*					
Age of DM onset (years)	54.95 ± 9.51	50.13 ± 8.13	54.28 ± 5.98	42.92 ± 8.74	.20
Male (%)	0.68 ± 0.48	0.43 ± 0.5	0.36 ± 0.49	0.33 ± 0.58	.10
Body mass index (kg/m^2^)	24.92 ± 3.71	25.16 ± 4.67	24.9 ± 2.61	22.48 ± 4.4	.84
Waist circumference (cm)	84.78 ± 7.5	85.59 ± 8.63	85.68 ± 7.9	83.5 ± 2.12	.91
Fasting plasma glucose (mmol/L)	10.48 ± 5.43	9.54 ± 3.63	9.72 ± 3.89	10.42 ± 3.60	.90
HbA1c (mmol/mol)	79.8 ± 13.7	90.6 ± 10.1	89.7 ± 7.9	83.6 ± 11.5	.5
Systolic blood pressure (mm Hg)	124.96 ± 16.33	127.85 ± 16.74	126.5 ± 18.75	134.33 ± 0.58	.50
Diastolic blood pressure (mm Hg)	72.5 ± 11.08	75.55 ± 11.52	74.64 ± 13.26	76 ± 12.53	.77
Pulse rate (BPM)	86.07 ± 13.62	88.73 ± 12.08	90.95 ± 11.26	83.67 ± 4.04	.34
Triglyceride (mmol/L)	1.37 ± 0.46	1.53 ± 0.61	1.42 ± 0.58	1.26 ± 0.58	.65
HDL (mmol/L)	1.34 ± 0.23	1.32 ± 0.33	1.31 ± 0.34	1.15 ± 0.23	.44
Calculated LDL (mmol/L)	3.07 ± 1.20	2.89 ± 1.06	3.24 ± 1.06	2.77 ± 0.51	.81
Direct LDL (mmol/L)	2.53 ± 0.61	3.23 ± 0.76	3.43 ± 1.18	N/A	.13
Creatinine (mmol/L)	**0.08 ± 0.02**	**0.06 ± 0.02**	**0.07 ± 0.02**	**0.07 ± 0.01**	.005*
eGFR (mL/min/1.73 m^2^)	**87.07 ± 19.67**	**102.68 ± 14.99**	**95.85 ± 17.52**	**101.33 ± 11.68**	.001*
*MSD cluster*					
Age of DM onset (years)	48.04 ± 10.31	53.58 ± 9.33	48.93 ± 10.02	36.81 ± 12.28	.06
Male (%)	0.6 ± 0.51	0.5 ± 0.51	0.46 ± 0.52	0.14 ± 0.38	.25
Body mass index (kg/m^2^)	**25.62 ± 4.2**	**28.18 ± 5.3**	**27.11 ± 4.49**	**30.85 ± 2.11**	.043*
Waist circumference (cm)	89.22 ± 12.48	90.46 ± 9.28	88.42 ± 9.29	103 ± 12.73	.34
Fasting plasma glucose (mmol/L)	9.03 ± 2.70	9.25 ± 4.54	9.14 ± 3.68	8.79 ± 3.05	.82
HbA1c (mmol/mol)	**72.7 ± 6.3**	**68.6 ± 2.7**	**64.8 ± 4.6**	**62.6 ± 3.4**	<.001**
Systolic blood pressure (mm Hg)	125.93 ± 11.5	135.95 ± 16.67	132.46 ± 15.26	136 ± 15.12	.25
Diastolic blood pressure (mm Hg)	73 ± 8.19	80.65 ± 9.87	77.69 ± 15.69	80 ± 14.58	.24
Pulse rate (BPM)	88.67 ± 13.44	86.11 ± 16.41	88.46 ± 11.79	96 ± 17.45	.35
Triglyceride (mmol/L)	3.42 ± 1.17	3.16 ± 1.53	3.47 ± 1.67	3.59 ± 1.40	.73
HDL (mmol/L)	1.02 ± 0.22	1.09 ± 0.34	1.07 ± 0.26	1.11 ± 0.29	.53
Calculated LDL (mmol/L)	2.35 ± 1.16	2.23 ± 1.13	2.57 ± 1.05	3.10 ± 1.57	.59
Direct LDL (mmol/L)	3.21 ± 0.96	3.65 ± 1.34	2.84 ± 0.77	N/A	.58
Creatinine (mmol/L)	**0.07 ± 0.02**	**0.09 ± 0.03**	**0.07 ± 0.02**	**0.06 ± 0.01**	.033*
eGFR (mL/min/1.73 m^2^)	**103.5 ± 17.71**	**83.37 ± 22.5**	**97.55 ± 15.9**	**113.14 ± 12.92**	.007*
*MOD cluster*					
Age of DM onset (years)	**40.53 ± 6.65**	**42.75 ± 7.2**	**41.04 ± 7.95**	**34.15 ± 8.1**	.012*
Male (%)	0.45 ± 0.5	0.26 ± 0.45	0.22 ± 0.42	0.36 ± 0.5	.17
Body mass index (kg/m^2^)	32.72 ± 6.63	32.9 ± 7.61	31.3 ± 4.65	34.75 ± 6.61	.63
Waist circumference (cm)	101.2 ± 20.83	104.18 ± 23.37	94.77 ± 11.6	96.33 ± 6.03	.55
Fasting plasma glucose (mmol/L)	**7.60 ± 1.76**	**9.22 ± 2.92**	**9.83 ± 3.07**	**8.87 ± 2.27**	.004*
HbA1c (mmol/mol)	**54.3 ± 11.6**	**65.7 ± 2.06**	**68.1 ± 7.0**	**68.7 ± 1.9**	.007*
Systolic blood pressure (mm Hg)	131.55 ± 12.09	130.84 ± 14.39	132.72 ± 14.83	131.82 ± 12.53	.77
Diastolic blood pressure (mm Hg)	77.74 ± 10.09	81.74 ± 11.3	80.84 ± 11.26	82.18 ± 13.76	.18
Pulse rate (BPM)	86.51 ± 13.39	92.92 ± 14.28	87.97 ± 12.38	91.82 ± 11.97	.42
Triglyceride (mmol/L)	1.86 ± 0.66	1.55 ± 0.55	1.69 ± 0.47	1.98 ± 0.86	.11
HDL (mmol/L)	1.15 ± 0.32	1.24 ± 0.28	1.24 ± 0.25	1.11 ± 0.25	.10
Calculated LDL (mmol/L)	2.71 ± 0.86	2.77 ± 1.11	3.05 ± 1.1	2.53 ± 0.66	.64
Direct LDL (mmol/L)	2.44 ± 0.80	3.61 ± 1.44	3.01 ± 0.91	N/A	.98
Creatinine (mmol/L)	0.07 ± 0.02	0.07 ± 0.02	0.06 ± 0.03	0.06 ± 0.01	.14
eGFR (mL/min/1.73 m^2^)	106.77 ± 13.36	100.62 ± 16.72	110.73 ± 13.88	114.71 ± 11.66	.09
*MARD cluster*					
Age of DM onset (years)	**62.26 ± 6.97**	**59.74 ± 6.85**	**58.58 ± 6.41**	**54.83 ± 4.32**	<.001**
Male (%)	0.41 ± 0.5	0.44 ± 0.5	0.38 ± 0.49	0.25 ± 0.5	.83
Body mass index (kg/m^2^)	26.63 ± 4.04	25.38 ± 4.41	26.54 ± 3.4	28.51 ± 3.25	.98
Waist circumference (cm)	91.4 ± 10.02	87.19 ± 10.5	93.4 ± 8.03	N/A	.61
Fasting plasma glucose (mmol/L)	7.21 ± 1.48	7.93 ± 1.99	7.44 ± 1.32	6.66 ± 1.54	.08
HbA1c (mmol/mol)	51.6 ± 11.3	53.3 ± 11.3	50.9 ± 13.2	52.8 ± 11.1	.77
Systolic blood pressure (mm Hg)	132.7 ± 14.71	131.69 ± 14.42	129.25 ± 12.21	130.25 ± 17.75	.17
Diastolic blood pressure (mm Hg)	73.7 ± 11.23	75.41 ± 11.85	75.13 ± 8.84	76.5 ± 11.68	.35
Pulse rate (BPM)	81.15 ± 12	81.3 ± 12.16	83.9 ± 13.48	91.5 ± 31.33	.67
Triglyceride (mmol/L)	1.56 ± 0.70	1.48 ± 0.68	1.56 ± 0.68	1.80 ± 0.82	.81
HDL (mmol/L)	1.40 ± 0.36	1.49 ± 0.37	1.42 ± 0.29	1.56 ± 0.17	.19
Calculated LDL (mmol/L)	2.64 ± 1.00	2.56 ± 0.85	2.69 ± 0.92	2.42 ± 0.99	.77
Direct LDL (mmol/L)	2.88 ± 0.77	3.16 ± 0.75	3.87 ± 0.40	N/A	.08
Creatinine (mmol/L)	0.08 ± 0.03	0.07 ± 0.02	0.07 ± 0.02	0.07 ± 0.02	.83
eGFR (mL/min/1.73 m^2^)	83.48 ± 17.51	85.26 ± 17.82	88.45 ± 14.35	87 ± 14.94	.49

DM: diabetes mellitus; eGFR: estimated glomerular filtration rate; MARD: mild age-related diabetes; SIDD: severe insulin-deficient diabetes; MSD: metabolic syndrome diabetes; MOD: mild obesity-related diabetes.

Data presented in mean ± standard deviation (S.D.). The bold text of p value is considered statistically significant (*p < .05 and **p < .001).

Multinomial logistic regression analysis showed the correlation between variables and the degree of relatives with diabetes, with (I) no relatives with diabetes used as reference (Figure 1(B)). Only one first-degree relative with diabetes and (II) had significantly decreased age at diagnosis (Beta = −0.031, *p* = .019) and increased DBP (Beta = 0.03, *p* = .008). Diastolic blood pressure was significantly increased in ≥2 relatives with diabetes with ≥1 first-degree relative with diabetes patients (III) (Beta = 0.026, *p* = .037). Patients who had ≥1 s-degree relative with diabetes but no first-degree relative with diabetes (IV) had the youngest age at the time of diabetes diagnosis (Beta = −0.077, *p* < .001).

## Discussion

Type 2 diabetes is a complex disease caused by the interaction between genetics and environmental factors [4]. Patients with type 2 diabetes and a FHD had a high risk of chronic complications, including diastolic dysfunction and microvascular complications [12]. However, it is unknown whether a different degree of FHD (which implies the role of genes) and its interaction with environmental factors such as diet, physical activity and socioeconomic status (which could be potential confounders) could influence the diverse natural history of the disease. Since we previously categorized newly diagnosed Thai type 2 diabetes into four clusters [7], we aimed to investigate whether there was a role for genetic factors that could affect these diverse type 2 diabetes clusters.

Our study participants were classified into four clusters including SIDD (19%), MSD (11%), MOD (25%) and MARD (45%). Each cluster had a significant difference in several anthropometric measurements, laboratory findings and especially age at the time of diagnosis. We also demonstrated the difference in PR and DBP among each group that was not mentioned in the previous study [13]. It was well established that many of these quantitative traits had diverse genetic determinants, including age at diagnosis [14], BMI [15], waist circumference [15], blood pressure [16], FPG [17], HbA1c [18], TG [19] and HDL level [20].

The finding that each cluster was significantly associated with the degree of FHD pointed to the role of genetic factors in the pathogenesis of these subgroups of type 2 diabetes. Moreover, each group had a distinct combination of degree of FHD which affected clinical characteristics, especially age at diagnosis both within and among each group. Ahlqvist and coworkers reported that a self-reported FHD of the first or second degree was the most common in MOD (87.8%) and SIDD (83.7%), but the least common in SAID (72.8%) [21]. Our study categorized FHD in more detail and demonstrated that more than 40% of SIDD had notably only first-degree relatives with diabetes, while approximately 13% of MSD and 10% of MOD had only second-degree relatives with diabetes. The association with second-degree relatives may reflect complex genetic contributions specific to these T2D subtypes, as well as intergenerational lifestyle factors or shared environmental exposures within extended family branches. This suggests that focusing on extended family history, rather than limiting it to first-degree relatives, could reveal valuable insights into the nuanced familial risk of certain T2D subtypes [22]. These findings highlighted the role of genetic heterogeneity leading to phenotypic heterogeneity. A previous study in rural Thailand showed that 60% of newly diagnosed type 2 diabetes had a first-degree relative with diabetes (maternal or paternal) resulting in an almost threefold risk of developing the disease [23]. However, in this study, we did not conduct a cluster analysis. A cross-sectional study of Han Chinese with newly diagnosed type 2 diabetes demonstrated that almost 70% had a FHD. It also showed that the age at diagnosis of type 2 diabetes was the lowest if both parents had the disease, followed by diabetes in the father, diabetes in the mother, and other types of diabetes family history, together with no FHD [24]. Vrijkotte and coworkers revealed that Dutch children with second-degree relatives with diabetes on both maternal and paternal sides had increased C-peptide levels compared with those who had either maternal or paternal and no FHD in second-degree relatives [25]. A study in a large cohort of multiple ethnic groups from a US research program showed a different probability of having type 2 diabetes among different types of self-report FHD in first-degree relatives OR: 7.49 (95% CI: 6.61–8.36; *p* < 2.0 × 10^−16^) if son or daughter had type 2 diabetes OR: 4.49 (95% CI: 4.25–4.72; *p* < 2.0 × 10^−16^) if father had type 2 diabetes, and OR: 3.96 (95% CI: 3.74–4.18) if mother had type 2 diabetes. In addition, FHD in first-degree relatives was associated with obesity OR: 2.04 (95% CI: 1.97–2.10) [26]. A study in Han Chinese indicated that different numbers of first-degree relatives with diabetes had diverse clinical characteristics but only the patients with a mother with diabetes were significantly associated with diabetic foot ulcers OR: 1.844 (95% CI: 1.022–2.154) [27]. We previously showed that each cluster of newly diagnosed type 2 diabetes had an unequal risk of developing diabetic retinopathy, nephropathy, and the requirement of insulin treatment [7]. In this study, we further demonstrated that each cluster had distinctive pattern of degree of FHD. Therefore, in Caucasians and Asians, a different degree of FHD could have diverse pathogenic consequences in the development of type 2 diabetes (different clusters, age at diagnosis, FPG, HbA1c), other related parameters (such as BP), even chronic complications and mode of treatment. Supporting this, a study on Alzheimer’s disease by Cannon-Albright et al. demonstrated that second and even third-degree relatives contribute to disease risk independently of first-degree relatives. The study found that Alzheimer’s risk increased significantly with second-degree relatives (RR 21.29 when combined with one first-degree relative) and even when only third-degree relatives were affected (RR 1.43). This finding underlines that inherited genetic factors for complex diseases like Alzheimer’s and possibly T2D have a broad influence extending beyond immediate relatives. Therefore, both in Alzheimer’s and in our T2D findings, it becomes clear that family history, including second-degree relatives, can provide a more complete picture of disease risk than a first degree-only approach [28]. These findings emphasize the importance of detailed FHD in classifying subtypes, especially newly diagnosed type 2 diabetes, early awareness of the development of its complications, and allocation of proper modes of treatment. Furthermore, it can create awareness that may enable lifestyle changes intended to decrease the risk of developing diabetes in other family members.

The strength of this study is, to our knowledge, the first cluster analysis of newly diagnosed type 2 diabetes in the Thai and Southeast Asian population and demonstrated that the variable in phenotypes could be influenced by various genetic factors, as implied by the findings that each cluster was associated with different degrees and combinations of FHD. Also, the discovering of association between FHD and T2D clusters might be information for a future larger scale of genomics study such as GWAS. The limitations are the relatively small number of subjects especially the MSD group. This study is a cross-sectional study from a single tertiary care hospital and self-reported FHD is prone to recall bias, sensitivity to wording, and inter-individual differences in knowledge of family history [29]. Future studies in larger and more balanced clusters are needed to validate whether FHD modifies the phenotypic expression or progression of specific diabetes subtypes and development of complications. Moreover, the potential for pooling data from similar studies enhances generalizability. Additionally, we did not explore FHD in more detail whether FHD was from paternal or maternal sites or a combination of them. Age at diabetes diagnosis in family members was not available. Furthermore, the genetic risk score (GRS) has been reported to be associated with FHD [30,31]. A study in the Finnish population showed that the polygenic risk score (PRS) and FHD are independent and provide complementary information on disease susceptibility [31]. They are not interchangeable. Although the PRS score was not incorporated into our study, we are working to identify the PRS and risk loci that may be associated with each cluster.

By demonstrating that different T2D clusters are associated with varying degrees and types of FHD, this study supports the notion that diabetes subtypes have distinct heritable components. Incorporating FHD into initial patient assessments may enhance the early identification of individuals at risk for more severe or early-onset diabetes phenotypes, particularly those within the SIDD cluster, who may benefit from earlier and more aggressive intervention strategies. Furthermore, recognizing the familial aggregation patterns in the MSD and MOD clusters may aid in identifying individuals who require lifestyle-focused interventions to address metabolic syndrome and obesity-related factors. On a broader scale, these findings could inform the development of targeted public health screening programs that prioritize high-risk families and enable more efficient allocation of preventive resources. Personalized treatment regimens could also be refined by aligning clinical management strategies with the patient’s cluster-specific risk profile and familial context.

## Conclusions

We demonstrated that in newly diagnosed type 2 diabetes patients in Thailand, a different degree and combination of FHD might have diverse effects on age at diagnosis, certain clinical characteristics/diabetic complications, laboratory parameters, and mode of treatment within and among each cluster. Therefore, genetic heterogeneity is responsible for phenotype heterogeneity, at least in newly diagnosed type 2 diabetes. More study with a larger sample size is needed to verify our findings. Moreover, the identification of the PRS that is associated with each cluster is necessary. Since PRS and FHD are not interchangeable and independent. They are complementary to each other and may lead to the discovery of genetic loci that are unique to each cluster. Studying these loci might lead to a better understanding of the pathogenesis of each cluster and suitable treatment and/or prevention of complications or precision medicine in the future.

## Data Availability

The data that support the findings of this study are available from the corresponding author upon reasonable request.
